# Differences in severity of cardiovascular anomalies in children with Noonan syndrome based on the causative gene

**DOI:** 10.3389/fped.2022.946071

**Published:** 2022-09-08

**Authors:** Nagham Shehade-Awwad, Yonatan Yeshayahu, Orit Pinhas-Hamiel, Uriel Katz

**Affiliations:** ^1^Pediatrics Department, Samson Assuta Ashdod Hospital, Ashdod, Israel; ^2^Noonan Multidisciplinary Clinic, Pediatric Endocrinology and Diabetes Unit, Edmond and Lily Safra Children's Hospital, Sheba Medical Center, Ramat-Gan, Israel; ^3^Faculty of Health Sciences, Ben-Gurion University, Beer Sheva, Israel; ^4^Sackler School of Medicine, Tel-Aviv University, Tel-Aviv, Israel; ^5^Pediatric Heart Institute, Edmond and Lily Safra Children's Hospital, Sheba Medical Center, Ramat-Gan, Israel

**Keywords:** genetics, Noonan syndrome, cardiovascular, genotype, phenotype, *PTPN11*, pulmonary stenosis

## Abstract

**Background:**

Noonan syndrome (NS) is a genetic syndrome, characterized by various dysmorphic features, cardiac anomalies, short stature, and developmental delay. NS is a leading cause of cardiovascular anomalies. The syndrome results from dysregulation in the RAS-MAPK pathway and is related to the RASopathy family syndromes. Pathogenic variants in more than 20 related genes have been identified in association with NS, and several genotype-phenotype correlations were suggested. The specific severity of the same cardiovascular anomalies has not been described as linked to a specific causative gene.

**Methods:**

For this retrospective, single-center study, data retrieved from medical charts of a multidisciplinary NS clinic included genetic diagnosis, cardiac malformations, the need for intervention, demographics, and prenatal diagnosis. We analyzed molecular genetics and the severity of cardiac malformations.

**Results:**

The cohort comprised 74 children with NS. Consistent with previous studies, pathogenic variants in *PTPN11* were the most common (62%). Cardiovascular anomalies presented in 57%; pulmonary stenosis (PS) was the most common (about 79% of anomalies). In children with pathogenic variants in *PTPN11*, PS tended to be more severe and required intervention in 53%, compared to 25% of children with PS and a variant in other genes.

**Conclusion:**

This first Israeli cohort of NS showed similar rates of cardiac malformations and genetic breakdown as previously published. Variants in *PTPN11* were prone to a higher risk for severe PS that requires intervention. This finding may assist in genetic counseling and cardiac treatment decisions, and stresses the importance of genetic in addition to clinical diagnosis of NS.

## Introduction

Noonan syndrome (NS) is a genetic disease characterized by distinctive craniofacial dysmorphic features, developmental delay, learning difficulties, short stature, and congenital cardiac anomalies (1, 2). Its prevalence is estimated as 1 in 1000 to 2500 live births (3, 4). NS is a heterogeneous condition, with variable phenotypic expression and severity (5). Clinical diagnosis is based on clinical criteria and a scoring system that includes the typical facial characteristics, typical heart defect, and short stature (6, 7). The syndrome is associated with pathogenic variants in multiple genes in the RAS-MAP kinases pathway, leading to dysregulation (8). NS shares a similar mechanism and overlapping clinical features with other syndromes such as: Noonan syndrome with multiple lentigines (previously called LEOPARD syndrome), cardiofaciocutaneous syndrome, and Costello syndrome. Collectively, these syndromes are referred to as RASopathies (9, 10). *PTPN11* is the most frequent causative gene in NS, which accounts for more than 50% of the patients (11). Various pathogenic variants in *PTPN11* have been identified, and attempts have been made to correlate them to syndrome severity (11, 12). More than 20 other genes related to NS have been discovered (13). Despite advances in genetic testing, many patients are diagnosed clinically without molecular testing to identify the causative variant (5).

NS is known as a leading cause of congenital heart disease (14). Pulmonary stenosis (PS) is the most common cardiac anomaly (50–60%), followed by hypertrophic cardiomyopathy (HCM) (20%) and secundum atrial septal defect (ASD) (6–32%) (15). Additional cardiac malformations, such as ventricular septal defect, peripheral PS, atrioventricular canal, aortic stenosis, mitral valve abnormalities, aortic coarctation, and coronary artery anomalies, are less common but have also been encountered (1, 16). Several studies that described phenotype-genotype correlations in NS focused on facial features relating to various genes or cardiovascular findings only within *PTPN11* variants (17–20).

To date, few studies have analyzed the correlation between cardiovascular severity and the indicated genes, or compared genotype with cardiovascular phenotype. The aim of our study was to characterize the cardiovascular features in a number of causative genes within a large cohort of patients with NS, and to describe the type and severity of the cardiovascular anomalies, as well as the need for intervention by catheter or surgery during infancy and childhood.

## Methods

The study was carried out at the multidisciplinary NS clinic, at Safra Children's Hospital, Israel, and was approved by the institutional Helsinki ethics committee.

A retrospective chart analysis was performed of children with NS who were followed in the clinic. Visits to specialty clinics were screened, including the cardiology and endocrine clinics; genetic test results and echocardiogram results were obtained. Inclusion criteria were children with a diagnosis of NS, either clinically or genetically. In the investigation of genotype-phenotype correlations, only those with a genetic diagnosis were included. The genetic diagnosis was based on ACMG variant classification guidelines (21).

Demographic data were described as means and standard deviations. Rates of cardiovascular malformations between genotypes were compared, and the need for intervention according to the causative genes was calculated as odds ratios and relative risks.

The diagnosis of HCM is established based on echocardiography. The hallmark finding is increased left ventricular (LV) wall thickening in the absence of a hemodynamic cause. In pediatric patients, measurements of LV wall thickness are normalized for age and body surface area using Z-score, which may be calculated by several methods (22–24). Herein, to compute the Z score for our patients with HCM, we used The Boston Hospital's Z score.

## Results

The study included 74 patients with NS; the mean age was 9.4 ± 6.03 years. In total of 40% were males; 15% had a parent who was diagnosed with NS. Fifty two patients had a molecular diagnosis and 22 had a clinical diagnosis only.

For 18 of the 52 patients with a molecular diagnosis, this was done by using various commercial RASopathy panels. 14 by exome sequencing (ES) and 4 by single-gene Sanger sequencing of *PTPN11* or *SOS1*. The mode of genetic testing was unavailable for the remaining 16 patients.

Of the 40 patients who had undergone prenatal screening tests, 85% (*n* = 34) had abnormal findings. Twenty patients showed increased nuchal translucency or a cervical cyst and 6 had polyhydramnios. Eleven patients had undergone fetal echocardiography, 7 of whom had abnormal findings.

Ten causative genes were found in our cohort; their prevalence are described in Table 1. Thirty two patients (62% of those with a genetic diagnosis) had a pathogenic variants in the *PTPN11* gene.

**Table 1:** The prevalence of gene pathogenic variants: (In total, 52 patients underwent genetic diagnosis).

**Gene variant**	**Prevalence, *n* (%)**
*PTPN11*	32 (62)
*SOS1*	6 (12)
*LZTR1*	3 (6)
*SHOC2, MAP2K, BRAF*	2 (4)
*NRAS, MRAS, KRAS, FRAS*	1 (2)

**Figure 1:**
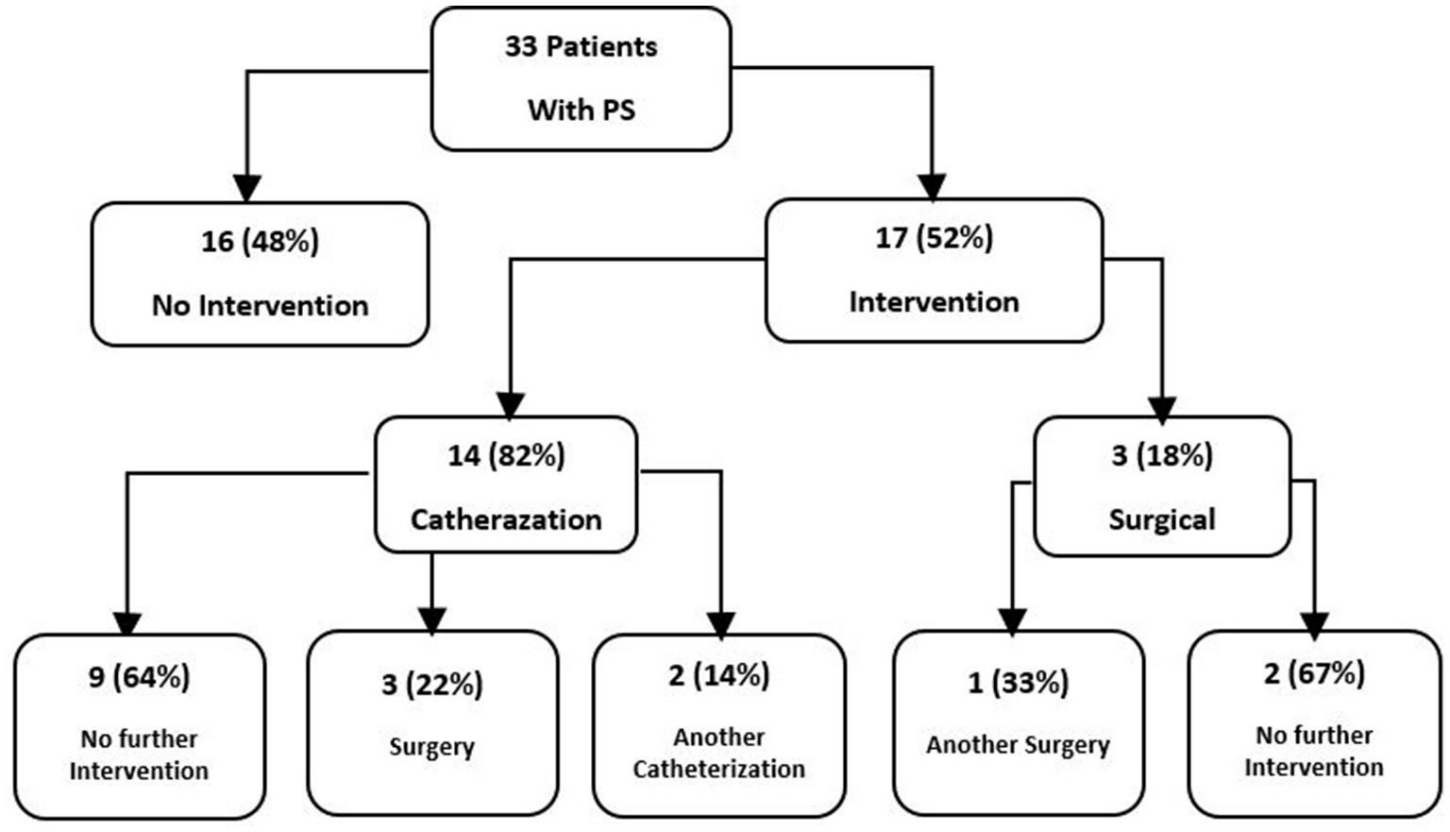
Intervention in 33 Noonan patients who has PS.

In total 57% (*n* = 42) of all the patients had a cardiac anomaly; of them, 71% (*n* = 30) had a single malformation and 29% (*n* = 12) had multiple cardiac malformations. The most common cardiac anomaly was PS, which accounted for 79% (*n* = 33) of the cardiac anomalies. Cardiovascular anomalies are summarized in Table 2. The patients with PS were classified according to its severity, as defined by the peak pulmonary valve gradient (Table 3). In total 52% (*n* = 17) of the patients with PS required intervention, either surgical (18%, *n* = 3) or catheterization (82%, *n* = 14). In total 35% (*n* = 6) of these patients required an additional intervention (Figure 1). In total 21% (*n* = 7) of patients with PS had a dysplastic valve, 5 of them required intervention.

**Table 2:** Cardiac anomalies in 42 patients with Noonan syndrome and with cardiac involvement.

**Cardiac defect**	**Number of patients**	**Percent**
Pulmonary stenosis	33	79
Hypertrophic cardiomyopathy	11	26
Atrial septal defect	5	12
Ventricular septal defect	2	5
Aortic stenosis	2	5
Dilated cardiomyopathy	1	2
Mitral regurgitation	1	2
Coarctation of aorta	1	2

**Table 3:** Pulmonary stenosis severity.

**PS severity**	**Peak pulmonary valve gradient (mmHg)**	**# of patients**	**Percent (%)**	**Mean age at diagnosis of Noonan syndrome (months)**
Mild	20–40	11	37	3.75
Moderate	40–60	7	23	29
Severe	>60	12	40	5.5

# indicates the number.

The second most common cardiac anomaly reported in our cohort was HCM, for which the prevalence was 26% (*n* = 11). In total of 64% (*n* = 7) were diagnosed during pregnancy or a short time after birth. The mean LV wall thickness Z score for our patients with HCM was 3.91. We calculated the mean LV mass index as 109.5 gr/m^2^ in males (*n* = 4); one of the females with a HCM diagnosis had available data for LV mass index, 69 gr/m^2^. These two values are considered above the normal range (>95 th percentile) (25, 26). Only 1 patient required septal myectomy. The third common cardiac anomaly in our cohort is ASD, counts for 12% (*n* = 5). Two of them required intervention.

The majority of patients (62%, *n* = 32) had pathogenic variants in the *PTPN11* gene; of them, 66% (*n* = 21) had cardiac anomalies. In total 81% of the latter (*n* = 17) were diagnosed with PS. In total 53% (*n* = 9) of patients with PS required intervention of either catheterization (*n* = 8) or surgery (*n* = 1). Mean age of intervention was 13 months. Three patients needed additional intervention; 2 required surgical and 1 required catheterization re-intervention. One patient required a third intervention.

In addition to PS, other less frequent cardiac anomalies were seen in the *PTPN11* group, as presented in Table 4. Three patients were diagnosed with both PS and HCM. This combination did not result in increased clinical severity and none of them required any intervention.

**Table 4:** Cardiac anomalies in 32 patients with Noonan syndrome, diagnosed with *PTPN11* variants.

**Cardiac anomaly**	**Number of patients**	**Percent**
Any cardiac anomaly	21	66
Pulmonary stenosis	17	81
Hypertrophic cardiomyopathy	5	24
Ventricular septal defect	2	10
Atrial septal defect	2	10
Aortic stenosis	1	5

As most patients (62%) had a pathogenic variant in the *PTPN11* gene, and the remainder were in small numbers (38%, *n* = 20), it was not possible to perform a subgroup analysis of every gene. Therefore, all those with pathogenic variants in genes other than *PTPN11* were clustered, and this non-*PTPN11* group was compared to the *PTPN11* group. In total 55% (*n* = 11) of the non-*PTPN11* group had a cardiac anomaly; of them, 73% (*n* = 8) were diagnosed with PS, and 25% (*n* = 2) required intervention of either catheterization (*n* = 1) or surgery (*n* = 1). Limited data were available regarding the age of intervention at this group with mean age of 6 months (*n* = 3). A subgroup analysis of the general prevalence of cardiac malformations and PS prognosis is shown in Table 5.

**Table 5:** Subgroup analysis of the general prevalence of cardiac malformations and the prognosis of pulmonary stenosis.

	***PTPN11* group** **(*n =* 32)**	***Non-PTPN11* group** **(*n =* 20)**	
Cardiac malformation	21 (66%)	11 (55%)	
Pulmonary stenosis (PS)	17 (81%)	8 (73%)	
**PS requiring intervention**	9 (53%)	2 (25%)	RR 2.08, OR 3.3 (CI 0.52–21)

We compared the LV wall thickness Z-score for the *PTPN11* and non-*PTPN11* groups. According to The Boston Z-score system, the mean Z-score for *PTPN11* patients was 0.07, and for the non-*PTPN11* group, 1.14.

We examined various variants in the *PTPN11* gene, in search of a correlation between the variant and the severity of cardiac anomalies in NS. Our analysis showed 7 patients with N308D pathogenic variant. In total 57% of them (*n* = 4) were diagnosed with cardiac defects; all had PS; 75% (*n* = 3) of the latter required intervention. Three patients had the M504V pathogenic variant in *PTPN11*. All of them were diagnosed with cardiac defects: 1 patient had PS, 1 had PS and HCM, and 1 had HCM and a ventricular septal defect.

## Discussion

This is the first description of an Israeli cohort of children with NS. The breakdown of causal genes was found to be similar to that previously reported. *PTPN11* pathogenic variants were the leading etiology, accounting for 62% of those with a genetic diagnosis, slightly higher than previously reported (50%) (1). *SOS1* variants were the second most frequent etiology, with a 12% prevalence. The rate of cardiac malformations was similar to that previously reported, including the rates of PS among those with cardiac anomalies. PS in patients with *PTPN11* variants tended to be more severe and required intervention in 53% of the patients, in contrast to only 25% of those with PS and non-*PTPN11* variants. This finding is different than previous descriptions of a 30% need for intervention in PS of NS, without describing the breakdown according to specific genes (1). Notably, the mean LV wall thickness was lower for patients with *PTPN11* variants than for those with non-*PTPN11* variants. These results are consistent with studies that showed a lower incidence of HCM with NS diagnosis and *PTPN11* variants (9). The findings highlight the importance of genetic diagnosis, to enable genetic counseling in early stages, and decision making by cardiologists. Given the large spectrum of clinical features within our cohort and in NS in general, any information that may be offered to patients and their families during early genetic diagnosis may help with decision making, and appropriate clinical follow up after delivery. Parents of 11 (15%) patients in our cohort were diagnosed with NS following their children's diagnosis.

Our cohort included children who were diagnosed over several years, during which the availability and funding of genetic testing changed significantly; this had a pronounced influence on the types of genetic testing performed. When funding and financial ability of parents were lacking, and the clinical picture was very clear, several parents decided to carry out a Sanger sequencing of *PTPN11* in early years. Later, when Noonan panels became more available and inexpensive, patients were recommended to undergo testing when a RASopathy was suspected. Finally, when the clinical picture was less clear, but some form of genetic syndrome was suspected, the most expensive test, namely ES, was ordered, and still funded by patients. This may explain the different ages at diagnosis observed between the three groups of children who underwent these three tests. Accordingly, the patients with the most clinically obvious signs were diagnosed earlier using Sanger sequencing, and the least obvious diagnosed later using ES. We expect that increasing availability and funding of ES will lead to earlier diagnosis of more subtle phenotypes of NS, including during prenatal screening.

Our study has several limitations. Firstly, due to the retrospective nature, the diagnosis algorithm varied, as it was carried out in a number of hospitals, and because many of our patients were referred to our specialized center following diagnosis. Therefore, some of the data were unavailable, and genetic testing differed between centers and time periods. Another limitation is that despite our relatively large sample, for a specific syndrome, especially of a small country, it was not large enough to enable statistical analysis of the clear trends observed. A multi-center or multinational study, combining multiple NS cohorts could enable subgroup analysis of the various genes, and of the various pathogenic variants within the same gene.

In summary, this is a first Israeli cohort of NS, reporting similar genetic breakdown and prevalence of cardiac anomalies as previously reported, but showing differences in severity according to gene variants. *PTPN11*-associated PS conferred a 2.1 relative risk for requiring intervention by surgery or catheterization, compared to PS in other gene variants. This stresses the importance of genetic testing, which enables more accurate prenatal counseling and medical decision making.

## Data availability statement

The raw data supporting the conclusions of this article will be made available by the authors, without undue reservation.

## Ethics statement

The study was carried out at the multidisciplinary NS-A clinic, at Safra Children's Hospital, Israel, and was approved by the institutional Helsinki Ethics Committee. Written informed consent was not required as per local legislation and institutional requirements.

## Author contributions

NS-A and YY contributed equally to all stages of this manuscript, including the study conception, Helsinki approval, data collection, and the writing of the manuscript. UK contributed to cardiac data interpretation. OP-H and UK contributed to manuscript revision, reading, and approving the submitted version. All authors contributed to the article and approved the submitted version.

## Conflict of interest

The authors declare that the research was conducted in the absence of any commercial or financial relationships that could be considered as potential conflicts of interest.

## Publisher's note

All claims expressed in this article are solely those of the authors and do not necessarily represent those of their affiliated organizations, or those of the publisher, the editors and the reviewers. Any product that may be evaluated in this article, or claim that may be made by its manufacturer, is not guaranteed or endorsed by the publisher.
